# Visual detection of the human metapneumovirus using reverse transcription loop-mediated isothermal amplification with hydroxynaphthol blue dye

**DOI:** 10.1186/1743-422X-9-138

**Published:** 2012-07-27

**Authors:** Xiang Wang, Qian Zhang, Fang Zhang, Fenlian Ma, Wenzhi Zheng, Zhihui Zhao, Yinglong Bai, Lishu Zheng

**Affiliations:** 1 State Key Laboratory for Molecular Virology and Genetic Engineering, National Institute for Viral Disease Control and Prevention, Chinese Center for Disease Control and Prevention, Ying-Xin Road 100, Xuan Wu District, Beijing, 100052, Peoples Republic of China; 2 Department of Children and Adolescent Health, China Medical University, North 2nd Road 92, Heping District, Shenyang, 110001, Peoples Republic of China

**Keywords:** Human metapneumovirus, RT-LAMP, RT-PCR

## Abstract

**Background:**

Human metapneumovirus (hMPV) is a major cause of acute respiratory infections ranging from wheezing to bronchiolitis and pneumonia in children worldwide. The objective of this study is to develop a visual reverse transcription loop-mediated isothermal amplification (RT-LAMP) assay for the detection of hMPV and applied to the clinical samples.

**Results:**

In this study, visual RT-LAMP assay for hMPV was performed in one step with the addition of hydroxynaphthol blue (HNB), and were used to detect respiratory samples. Six primers, including two outer primers (F3 and B3), two inner primers (FIP, BIP) and two loop primers (LF and LB), were designed for hMPV N gene by the online software. Moreover, the RT-LAMP assay showed good specificity and no cross-reactivity was observed with human rhinovirus (HRV), human respiratory syncytial Virus (RSV), or influenza virus A/PR/8/34 (H1N1). The detection limit of the RT-LAMP assay was approximately ten viral RNA copies, lower than that of traditional reverse transcriptase polymerase chain reaction (RT-PCR) 100 RNA copies. In the 176 nasopharyngeal samples, 23 (13.1%) were conformed as hMPV positive by RT-LAMP, but 18 (10.2%) positive by RT-PCR.

**Conclusion:**

Compared with conventional RT-PCR, the visual hMPV RT-LAMP assay performed well in the aspect of detect time, sensitivity, specificity and visibility. It is anticipated that the RT-LAMP will be used for clinical tests in hospital or field testing during outbreaks and in emergency.

## Background

Osterhaus first reported and named the human metapneumovirus (hMPV) [1]; it was also detected in Europe, America, Australia, and other countries [2-4]. In 2003, hMPV was reported in China [5]. hMPV is a negative-sense, single-stranded RNA virus belonging to the genus *Metapneumovirus* in the subfamily *Pneumovirinae* of the family *Paramyxoviridae*. It often co-infects patients and causes acute respiratory diseases in different age groups together with other respiratory virus, and severe cases are suffered mostly by infants, the elderly, and patients with heart and lung diseases [6-9].

Currently, the most common hMPV detection methods are by virus isolation, enzyme-linked immunoassays, McAb immuno-fluorescent assays and molecular biology methods based on the polymerase chain reaction (PCR, RT-PCR, nested PCR, and real-time PCR). However, sensitive cell lines for hMPV are limited. The cell line LLC-MK2 can reach a 100% infection within 5 days of isolation [10], but this is far from fulfilling the requirements in terms of time efficiency for positive identification during outbreaks. In comparison, hMPV antigen detection requires the preparation of monoclonal antibodies; this entails significant costs and can be time-consuming. Although RT-PCR, qRT-PCR, and microarrays are suitable for high-throughput analyses and can produce rapid, specific, and sensitive results, the expensive instruments required for such tests restrict their application.

LAMP is a novel nucleic acid isothermal amplification technique developed by Notomi [11]. LAMP relies on autocycling strand-displacement DNA synthesis performed by Bst DNA polymerase. Additionally, RNA can be amplified by reverse transcription coupled with LAMP (RT-LAMP) in one step, making it suitable to detect RNA viruses such as hMPV. RT-LAMP employs a set of six specially designed primers that recognize eight distinct sequences of the target, and only when three pairs of specific primers match the target sequence exactly can the gene amplification start. The whole reaction can be completed in 1.5 h and the amplified product shows a ladder-like pattern by agarose gel electrophoresis. Moreover, this method produces a large amount of amplified product, resulting in easier detection by visual judgment according to the turbidity of the reaction mixture. Therefore, LAMP and RT-LAMP method have been used as a powerful gene amplification tool due to its high specificity and sensitivity under isothermal condition.

In this study, we established a RT-LAMP assay to detect hMPV, and the amplification result can be observed by hydroxynaphthol blue (HNB) dye-mediated visualization using the naked eye. Compared with conventional RT-PCR, the newly established visual RT-LAMP assay is simple, efficient, cost-effective and convenient that could be used as a diagnostic tool in clinical respiratory samples.

## Results

### Sequencing and restriction endonuclease digestion of RT-LAMP products

**Figure 1:**
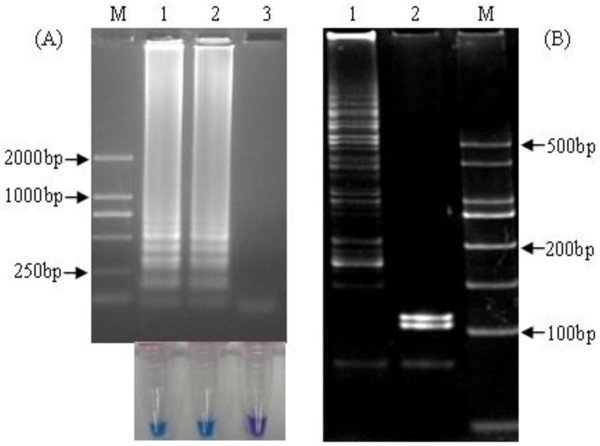
**hMPV RT-LAMP amplification and digestion of positive RT-LAMP products. (A**) RT-LAMP products were examined by agarose gel electrophoresis (upper panel) and visually amplification for color change (lower panel). Lane M, DNA marker DL2000 (Biomed, China, with bands of 2000, 1000, 750, 500, 250 and 100 bp); 1–2, positive hMPV RNA; 3, DEPC-treated H_2_O. **(B)** RT-LAMP products were digested with Msp I, and two fragments (103 bp, 116 bp) were observed by polyacrylamide gel electrophoresis attained with ethidium bromide and photographed under a UV transilluminator. 1, RT-LAMP products without digestion; 2, RT-LAMP products digested by Msp I; M, DNA marker DL500 (Biomed, China, with bands of 500, 400, 300, 250, 200, 150, 100 and 50 bp).

As shown in Figure 1A, RT-LAMP products of hMPV RNA showed a ladder-like pattern upon agarose gel electrophoresis. When the sample tube did not contain target RNA, no amplification was seen. In addition, The amplification products can be observed by naked eyes under the natural light. The negative control reaction showed violet, but the color became sky blue for the positive amplification. Sequencing results of the recombinant plasmid pGEM-T-N217 indicated that the 217 bp target sequence was 100% homologous to the sequence of hMPV N gene used for the primers design (GenBank EF081360) (not shown).

By sequence analysis, there exists a unique Msp I restriction site in the F1 region. After digestion of RT-LAMP products with the Msp I overnight, the 103 and 116 bp fragments were observed, and were in good accordance with those predicted theoretically from the expected structures. The results of sequence and digestion confirmed that the products were amplified specificly from the hMPV target region, (Figure 1B).

### Specificity and sensitivity of the hMPV RT-LAMP

**Figure 2:**
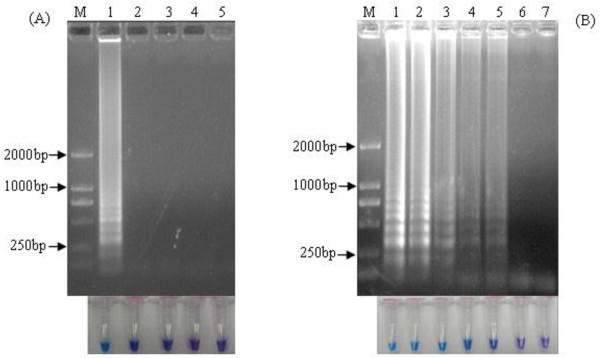
**Specificity and sensitivity of hMPV RT-LAMP. (A)** HRV, influenza virus A/PR/8/34 (H1N1), and RSV positive nucleic were amplified by RT-LAMP using the primers as in Figure 4. The RT-LAMP products were tested by agarose gel electrophoresis (upper panel) and direct color change (lower panel). Lane M, DNA marker DL2000 (Biomed, China, with bands of 2000, 1000, 750, 500, 250 and 100 bp); 1, positive hMPV RNA; 2, influenza virus A/PR/8/34; 3, RSV; 4, HRV; 5, DEPC-treated H_2_O. **(B)** RNA serially diluted was amplified and the RT-LAMP products were tested by two methods as above. Lane M, DNA marker (as above); 1, 10^5^ copies; 2, 10^4^ copies; 3, 10^3^ copies; 4, 10^2^ copies; 5, 10 copies; 6, Single copy; 7, DEPC-treated H_2_O.

HRV, influenza virus A/PR/8/34 (H1N1), and RSV viral nucleic acid were amplified by RT-LAMP method using 3 pairs of primers of hMPV. No cross-reactivity was detected with the other three respiratory viruses, indicating a high level of specificity of the RT-LAMP assay for hMPV (Figure 2A). Positive color (sky blue) was only observed in the preparation of the hMPV, whereas none of the other viruses showed a color change.

In Figure 2B, lanes 1–5 showed that decreases in the amount of nucleic acids (10^5^–10 copies/reaction) generated fading amplified band signals. Ten or more copies of the nucleic acid resulted in RT-LAMP-specific ladder-like bands, indicating a sensitivity level of at least 10 copies needed for amplification.

### RT-PCR assay and its sensitivity

**Figure 3:**
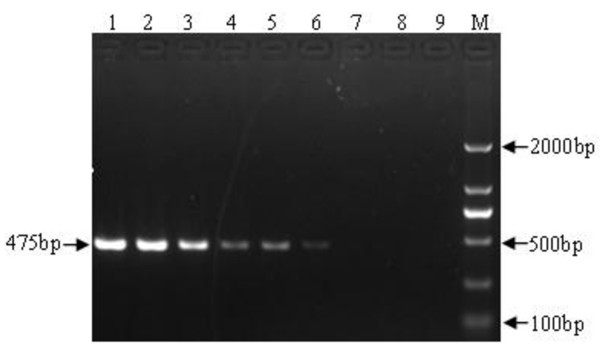
**Sensitivity of RT-PCR assay.** RNA was diluted serially and was amplified by RT-PCR, and products were examined by agarose gel electrophoresis. 1, 10^4^ copies; 2, 5000 copies; 3, 10^3^ copies; 4, 500 copies; 5, 200 copies; 6, 100 copies; 7, 10 copies; 8, 1 copies; 9, DEPC-treated H_2_O; Lane M, DNA marker DL2000 (Biomed, China, with bands of 2000, 1000, 750, 500, 250 and 100 bp).

RT-PCR products (3ul) were subjected to electrophoresis on 1% agarose gel stained with ethidium bromide, and a 457 bp specific band could be seen clearly. As shown in Figure 3, RT-PCR assay could detect as little as 100 copies of RNA molecules, indicating less sensitive than the RT-LAMP method.

### Specimen testing and comparative assay sensitivity

To evaluate the clinical sensitivity of the RT-PCR assay and RT-LAMP assay under routine conditions, 176 nasopharyngeal specimens obtained from children with respiratory tract infections was chosen for investigation using both assays. 23 (13.1%) of these specimens were positive by the RT-LAMP assay, but 18 (10.2%) samples were identified hMPV positive by RT-PCR screening assay. Additionally, all RT-PCR positive specimens could be detected with RT-LAMP. This result suggested that RT-LAMP method was more feasible than RT-PCR when detecting hMPV from clinical samples, because of the higher sensitivity of RT-LAMP.

## Discussion

Loop-mediated isothermal amplification assay (LAMP) was originally designed for genetic testing and applied to viruses and other pathogens [11-13]. Using six primers that target eight distinct gene sequences, the reaction is initiated only if all the primers match with the targets sequence correctly. Thus, these assays offer high levels of specificity, selectivity, sensitivity, and efficiency. In particular, reverse transcription and amplification reactions of RNA viruses can be completed in the meantime under isothermal environments, saving time and reducing experimental error.

The most conserved segment within the N gene (GenBank EF081360) was selected as the target. The entire testing process under isothermal conditions of 65°C takes only 1.5 h (the DNA template must be pre-denatured at 95°C prior to the isothermal reaction). Additionally, results from the LAMP can be readily observed by the white precipitate of magnesium pyrophosphate generated during the reaction. Just because no laboratory equipment is needed for the determination of results, RT-LAMP can easily be used in field-testing. Moreover, A real-time monitoring turbidimeter was developed to monitor LAMP reactions [14]. Through continuous exploration, LAMP has been combined with a fluorescent dye for quantification in real-time reactions [15,16]. For example, a metal ion indicator, hydroxynaphthol blue (HNB), was added to LAMP assay [17-19]. The HNB dye-based assay has a remarkable advantage compared with other color-based assays in that HNB is mixed prior to amplification. Thereby, the reaction tubes need not to be opened, which reduce the risk of cross-contamination.

LAMP technology has been applied to a variety of nucleic acid detection assays for pathogens, such as H5N1-HPAIV, HBV, HIV-1, MTB, O157, trypanosomes, and plasmodium [15,16,20-23]. This study established an RT-LAMP method with HNB dye to detect the hMPV N gene, which has significantly higher sensitivity (10 copies) levels than the conventional RT-PCR method and did not cross-react with RSV, HRV, or A/PR/8/34 (H1N1) viral nucleic acids. Restriction enzyme and sequence analyses also validated its specificity.

In the testing of clinical respiratory samples, 18 hMPV positive samples were detected by the traditional RT-PCR method, but 23 hMPV positive samples by RT-LAMP method due to the higher sensitivity. The epidemiological analysis indicated that children with HMPV varied from 20 days to 146 months of age, and the majority of patients (82.6%, 18/23) were aged at under ≤5 years. The main clinical diagnoses included bronchopneumonia (52.2%, 12/23), acute respiratory tract infection (26.1%, 6/23), acute asthmatic bronchopneumonia (17.4%, 4/23) and pneumonia (4.3%, 1/23). In short, RT-LAMP with HNB dye was shown to be a sensitive and easy assay for detection of hMPV. Given these benefits, RT-LAMP is considered a useful and promising technique for the less equipped primary sector. It can also play an important role in the early diagnosis and control of diseases during outbreaks in remote areas or under field conditions.

## Conclusion

In brief, the RT-LAMP assay is a simple, rapid, and accurate nucleoside amplification method. In combination with HNB dye, the visual RT-LAMP is much easier for the result determination, and this method can be used for the diagnosis of hMPV not only in the laboratory but also in the field.

## Methods

### Viral nucleic acid samples and clinical samples

HRV, hRSV, influenza virus A/PR/8/34 (H1N1), hMPV nucleic acid samples extracted from hMPV positive respiratory specimen were provided by the Gene Laboratory (China CDC). In 2009, 176 nasopharyngeal specimens from 176 children younger than 14 years of age with acute respiratory infection, who had been admitted to the 8^th^ People’s Hospital of Shenyang, Liaoning Province, China, were collected. The samples were transported to the laboratory in China CDC, and stored at –80°C until further processing. Informed consent was obtained from the children’s parents or guardians. This study was approved by the Ethical Review Committee of National Institute for Viral Disease Control and Prevention, China CDC (No. 200806).

### Primer design

**Figure 4:**
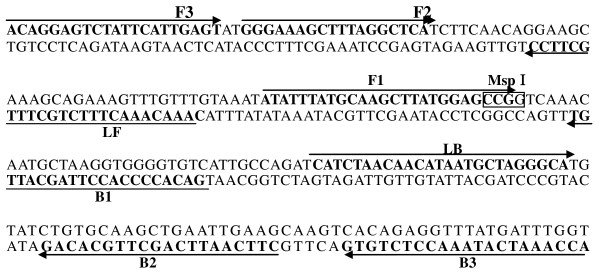
**hMPV N gene RT-LAMP primer design and target sequence location.** Genomic DNA sequence of hMPV N gene (GeneBank Accession number: EF081360) from 659 to 875 nt in orientation 5’ to 3’. The primers sequences are highlighted with Italic and bold, and the arrows show the primers direction from 5’ to 3’.

Several hMPV genomic sequences isolated in China were downloaded from GenBank and aligned with each other using the AlignX function in Vector NTI Advance 10 (Invitrogen). The most conserved segment within the N gene of hMPV/CS038 N gene (GenBank EF081360) was selected as the target. All LAMP primers were designed by the online software http://primerexplorer.jp/e/ (Eiken Chemical Co., Ltd., Tokyo, Japan). Six primers were used for the RT-LAMP assay, including two outer primers (F3 and B3), a forward inner primers FIP (F1c-F2), a backward inner primer BIP (B1c-B2), and two loop primers (LF and LB). The location and sequences of each primer were shown in Table 1 and Figure 4. The predicted length of RT-LAMP is 217 bp.

**Table 1:** Primer sets designed to detect hMPV N gene by RT-LAMP

**Primer**	**Sequence**	**Genome position**	**Length**
F3	ACAGGAGTCTATTCATTGAGT	659–679	20
B3	ACCAAATCATAAACCTCTGTG	855–875	18
FIP(F1c + F2)	CGGCTCCATAAGCTTGCATAAATAT-GGGAAAGCTTTAGGCTCA	(739–763) + (682–699)	22 + 20
BIP(B1c + B2)	ACAATGCTAAGGTGGGGTGTC-CTTCAATTCAGCTTGCACAG	(770–789) + (830–849)	20 + 20
LF	CAAACAAACTTTCTGCTTTGCTTCC	709–733	20
LB	CATCTAACAACATAATGCTAGGGCA	800–824	25

### RT-LAMP reaction

The RT-LAMP reaction was performed in a total volume of 25 μL mixture, containing 5 μM F3 and B3 (1 μL each primer), 40 μM FIP and BIP (1 μL each primer), 20 μM LF and LB (1 μL each primer), 10 mM dNTPs (2.5 μL), 25 mM betain (1 μL), 150 mM MgSO_4_ (1 μL), 10× Bst buffer (2.5 μL), 8 U/μL Bst DNA polymerase (1 μL) (New England Biolabs), 10 U/μL AMV reverse transcriptase (1 μL) (Invitrogen), 3 mM HNB (1 μL), template RNA (1 μL) and double-distilled water (ddH_2_O; 8 μL). The mixture was incubated at 65°C for 1.5 h and then heated at 80°C for 5 min to terminate the reaction. Template RNA was replaced by ddH_2_O as negative control. A portion of each product was analyzed by 1% agarose gel electrophoresis stained with ethidium bromide and photographed under a UV transilluminator. In addition, the amplification product also can be visually inspected for the color change from violet to sky blue, and the negative control reaction should have remained violet. The tubes were observed by naked eyes and photographed under the natural light.

### Sequencing of RT-LAMP products

After electrophoresis, DNA bands about 217-bp obtained from the RT-LAMP positive gel was extracted using a Gel/PCR DNA Fragments Extraction Kit (Geneaid), and was amplified specially by PCR method with the sense and antisense primers F3 and B3. Amplification was performed as follows: 94°C for 8 min, and then 40 cycles of 94°C for 30 s, at 50°C for 30 s, 72°C for 40 s, with a final 72°C extension for 10 min. The PCR was carried out in a total volume of 30 μL containing 10 μM F3 and B3 (1 μL each primer), 10 mM dNTPs (1 μL); 10× PCR buffer (3 μL), 5 U of rTaq (1 μL), extracted products (5 μL), and ddH_2_O (18 μL). The 217-bp product was extracted after 1% agarose gel electrophoresis, cloned into pGEM-T Easy Vector (Promega), and then transformed into competent DH5α cells (BioMed). The recombinant plasmid pGEM-T-N217 was extracted from positive clones was sequenced by Invitrogen (China).

### Restriction enzyme digestion analysis of RT-LAMP products

RT-LAMP products were digested with Msp I in a 20 μL reaction containing Msp I (1 μL) (Takara), 10 × Msp I Basal Buffer (2 μL), 0.1% bovine serum albumin (2 μL), LAMP products (5 μL), and ddH_2_O (10 μL), incubated at 37°C overnight, after which the DNA band pattern were analyzed on 12% polyacrylamide gel electrophoresis stained with ethidium bromide and photographed as above.

### RT-LAMP specificity test

To evaluate the cross-reactivity of the hMPV N gene primer set with other common respiratory viruses, viral nucleic acid extracted from HRV, hRSV, and influenza virus A/PR/8/34 (H1N1) strains were tested and a DEPC-treated water as negative control under the same conditions.

### RT-LAMP sensitivity test

To obtain the detection limit of the RT-LAMP assay, the recombinant plasmid pGEM-T-N217 linearized by the vector-specific restriction enzyme Nde I was transcribed *in vitro* using the T7 RiboMAX Express Large Scale RNA Production System (Promega) and digested with RNase-Free DNase Set (Qiagen). Then the RNA was purified using an RNeasy Mini Kit (Qiagen) according to the manufacturer’s protocol and the concentration (μg/mL) was determined by Biophotometer (Eppendorf). The RNA copy number was calculated using the following formula: number of copies (copies/μL) = amount (ng) × 6.022 × 10^23^/length (base pairs) × 10^9^ × 340. 10-fold serial dilutions of RNA ranging comprising 10^5^ to a single copy were applied to determine RT-LAMP sensitivity limit.

### RT-PCR detection and sensitivity test

hMPV cDNA was obtained from viral nucleic acid samples using QIAGEN OneStep RT-PCR kit (Invitrogen) according to the manufacturer’s instructions. We designed RT-PCR primers for hMPV N gene (forward: 5’-CAACATTCCGCAGAACCAGC -3’ and reverse: 5’-GCCCATTTCACGCACCAGA -3’). Amplification was performed as follows: 50°C for 30 min, 95°C for 15 min, and then 40 cycles of 94°C for 30 s, at 57°C for 30 s, 72°C for 45 s, with a final 72°C extension for 10 min. Moreover, in order to evaluate the sensitivity of the RT-PCR assay, 457-nucleotide PCR product was extracted, cloned to pMD18-T vector (Takara), transcribed to RNA, and finally analyzed the sensitivity of RT-PCR. All the process and condition was similar to the sensitivity assessment for RT-LAMP described as above.

### Testing of clinical respiratory specimens by RT-LAMP and RT-PCR

To compare the availability of RT-PCR method and RT-LAMP method, 176 nasopharyngeal samples of the 176 children with acute respiratory tract infections were extracted for RNA using the Viral Nucleic Acid Extraction Kit (Geneaid) and were tested using visual RT-LAMP and RT-PCR methods developed as above.

## Competing interests

The authors declare that they have no competing interests.

## Authors’ contributions

XW and LSZ designed this study; XW, QZ, FZ, WZZ, FLM performed this study; YLB and ZHZ collected the clinical specimens; WX and QZ wrote the paper; QZ, YLB and LSZ revised the manuscript critically. All authors read and approved the final manuscript.
